# Effects of bone marrow aspirate concentrate and platelet-rich plasma on patients with partial tear of the rotator cuff tendon

**DOI:** 10.1186/s13018-017-0693-x

**Published:** 2018-01-03

**Authors:** Sang Jun Kim, Eun Kyung Kim, Sun Jeong Kim, Da Hyun Song

**Affiliations:** Department of Physical and Rehabilitation Medicine, Stem Cell & Regenerative Medicine Institute, Samsung Medical Center, Gangnamgu, Irwonro, 81th street, Seoul, 135-710 South Korea

**Keywords:** Bone marrow aspirates, Platelet-rich plasma, Rotator cuff tear, Degeneration

## Abstract

**Background:**

We compared the clinical course of rotator cuff tears between rotator cuff exercise and bone marrow aspirate concentration (BMAC)-platelet rich plasma (PRP) injection to identify the therapeutic effects of BMAC-PRP on partial tear of the rotator cuff tendon.

**Methods:**

Twenty-four patients with partial tear of the rotator cuff tendon participated in this study. Twelve patients underwent extraction of BMACs and PRP and received the injection of BMAC-PRP at the tear site under ultrasound guidance. Twelve patients in the control group were asked to perform the rotator cuff exercise for 3 months. Visual analog scale (VAS) and manual muscle test (MMT) scores of the supraspinatus muscle were measured, and the American Shoulder and Elbow Surgeons (ASES) score was recorded before, 3 weeks, and 3 months after injection. Tear size was measured by the greatest longitudinal tear length.

**Results:**

The change in the VAS differed between groups at 3 months (*P* = 0.039) but not at 3 weeks (*P* = 0.147). The ASES scores in the BMAC-PRP group changed from 39.4 ± 13.0 to 54.5 ± 11.5 at 3 weeks and 74.1 ± 8.5 at 3 months while those in the control group changed from 45.9 ± 12.4 to 56.3 ± 12.3 at 3 weeks (*P* = 0.712) and 62.2 ± 12.2 at 3 months (*P* = 0.011). The tear size decreased at 3 weeks or 3 months after the BMAC-PRP injection but was not significantly different from that in the control group.

**Conclusions:**

BMAC-PRP improved pain and shoulder function in patients with partial tear of the rotator cuff tendon.

**Trial registration:**

The patients were registered in the institutional board registry of Samsung Medical Center (registry number 2014-07-173).

**Electronic supplementary material:**

The online version of this article (10.1186/s13018-017-0693-x) contains supplementary material, which is available to authorized users.

## Background

Rotator cuff tendon tear is a common shoulder problem with a prevalence of about 21% in the general population [1]. The rotator cuff tendon tear is multifactorial disorders caused by age-related degeneration, oxidative stress, and vascular changes although the pathophysiology of rotator cuff tear is not fully understood [2, 3]. The risk factors include traumatic injury, degenerative changes, repetitive impingement, genetic predisposition, and smoking [1, 4].

Rotator cuff tendon tear is treated by non-surgical and surgical methods. Non-surgical treatments are rotator cuff strengthening exercise, oral medication, and corticosteroid injection. However, all of these focus on symptomatic treatments rather than restoration of shoulder function and prevention of the progression of tears. Rotator cuff stretching and strengthening exercise can improve shoulder function, but the protocols are not standardized and its effect is not evident yet [5]. The natural course of non-surgically treated rotator cuff tears is anatomic tear deterioration, not spontaneous regeneration in the majority of patients [6, 7]. Surgical repair of the torn rotator cuff can restore shoulder function and prevent the progression of tear, but failure rates of rotator cuff tear after surgical repair range from 0 to 78% [8].

Biological adjuvants, such as platelet-rich plasma (PRP) and stem cells, have been used to enhance the regeneration of torn rotator cuffs and improve symptoms in preclinical studies [9–11]. PRP and stem cells enhance the regenerative potentials of tendon stem cells [12–14] and regeneration of torn supraspinatus in preclinical studies [15, 16]. Several clinical studies have described PRP-augmented rotator cuff repairs in patients with rotator cuff tears [17, 18]. Therefore, regenerative biological treatment will be helpful to restore shoulder function and prevent the progression of tear.

Bone marrow mesenchymal stem cells (BMMSCs) and bone marrow aspirate concentrates (BMACs) are good therapeutic candidates for use in repair of damaged structures [19], but adequate scaffolds are needed because of inadequate survival, reparative capacity, and differentiation capacity of BMMSCs and BMACs without them [20]. In addition to its own regenerative potentials, PRP can play a role in scaffolds of BMAC, and BMAC-PRP complexes have shown good regenerative potential in diabetic ulcers [21], osteochondral defects [22], and spinal cord injury [23].

We hypothesized that a BMAC-PRP complex would enhance regeneration of torn rotator cuff tendons and improve clinical symptoms. We compared the clinical course of partial tear of the rotator cuff tendon between rotator cuff exercise and BMAC-PRP injection to identify the therapeutic effects of BMAC-PRP on partial tear of the rotator cuff tendon.

## Methods

This study was conducted as a prospective, non-randomized comparative, single-blind study. Patients who visited the outpatient department in our hospital from August 2014 to December 2016 and were diagnosed with partial tear of the rotator cuff tendon were included in our study. Inclusion criteria were (1) no history of shoulder surgery during the past 3 months, (2) no abnormal findings on simple radiography, (3) partial tear of the rotator cuff tendon diagnosed with ultrasound or magnetic resonance images, (4) no abnormalities in blood coagulation and routine laboratory examination, (5) no history of steroid injection during the past 3 months, and (6) no history of malignancy. Exclusion criteria were (1) history of shoulder surgery within 3 months, (2) presence of osteophyte or bony deformity on simple radiography, (3) complete tear of the rotator cuff tendon, (4) presence of abnormality in blood coagulation, complete blood count, or blood chemistry, (5) positive urine pregnancy test in case of fertile woman, (6) recent steroid injection within 3 months, or (7) history of malignancy.

The patients had experienced shoulder pain for more than 2 months, and they felt any improvement neither by oral medication nor by physical modalities.

Partial tear of the rotator cuff tendon was diagnosed when the rotator cuff tendon showed irregular hypo-echogenicity or anechogenicity without retraction or atrophy of the cuff in the sonographic findings. We excluded the patients with retraction or atrophy of the cuff from our study because the tear size could not be measured and the tear site for the injection could not be defined.

A total of 24 patients participated in this study; 12 patients were assigned to the BMAC-PRP group at first, and 12 patients were assigned to the control group. Sample size was calculated based on the difference of American Shoulder and Elbow Surgeons (ASES) scores at 12 weeks in a previous study [24] with a power of 80% on a 5% significance level. Patients were assigned to the control group sequentially after recruitment was completed for the BMAC-PRP group. Demographic data including age, sex, and symptom duration were collected.

**Fig. 1:**
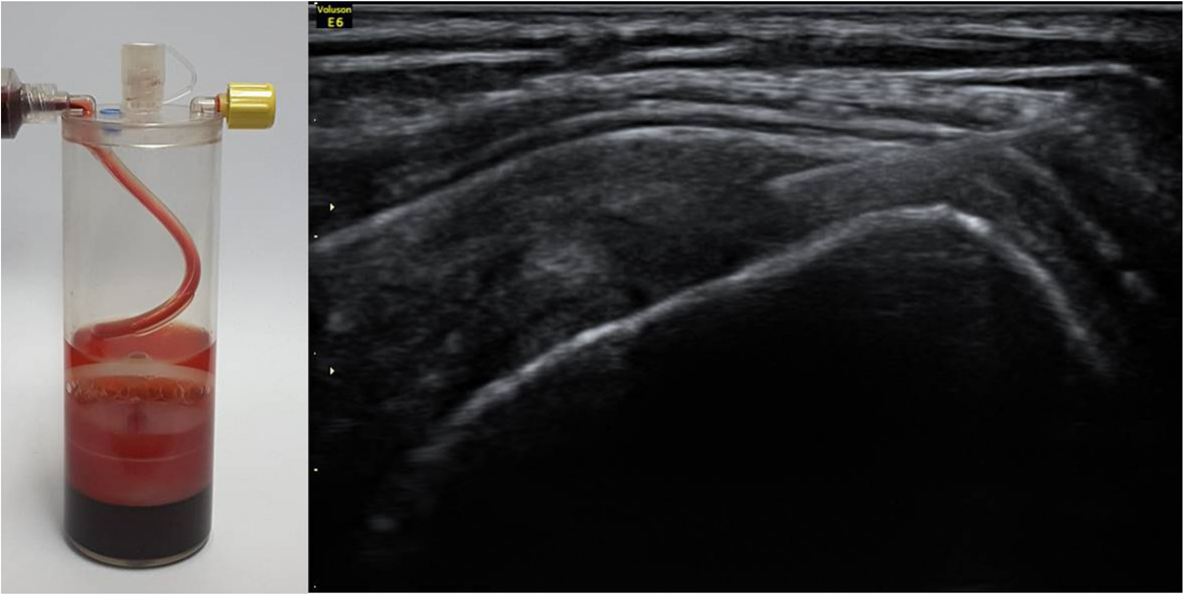
Bone marrow aspirate concentrates (BMAC) were acquired from the bone marrow and centrifuged with a BIOMET MarrowStim™ Mini kit to isolate the concentrated BMACs (left side). The patients received the BMAC-PRP injection at the tear site under ultrasound guidance by one experienced physiatrist (right side)

Patients in the BMAC-PRP group underwent extraction of their BMACs and PRP. For the extraction of BMACs, the patient lay on the table in a prone position. After the iliac crest was localized by palpation, the area was sterilized by povidone iodine, and the skin and periosteum were anesthetized by using 1% lidocaine. A bone marrow aspiration needle penetrated into the iliac bone and progressed to the bone marrow site. Bone marrow aspirates were acquired from the bone marrow and centrifuged with a BIOMET MarrowStim™ Mini kit (Biomet Biologics, Inc., Warsaw, IN, USA) to isolate the concentrated BMACs (Fig. 1). Peripheral blood (30 ml) was acquired from the left antecubital vein and was centrifuged with a BIOMET GPS™ III kit (Biomet Biologics, Inc.) to extract the PRP. After acquiring BMACs and PRP, 2 ml BMACs were mixed with 1 ml of PRP in a 5-ml syringe. The patients received the BMAC-PRP injection at the tear site under ultrasound guidance by one experienced physiatrist (Fig. 1). They did not receive any physical therapy to confirm the therapeutic effect of BMAC-PRP.

Patients in the control groups were taught rotator cuff exercise by an experienced physical therapist, and they were asked to perform the program daily on their own for 3 months. Rotator cuff exercise comprised of stretching, scapular stabilization exercise, and strengthening exercise. The specific exercise protocols were presented in the Additional file 1.

Visual analog scale (VAS) and manual muscle test (MMT) scores of the supraspinatus muscle were measured by one experienced physician who was blind to the group assignment of each patient. Manual muscle test was performed at 90° abduction state of the glenohumeral joint and 30° adduction state of the scapular plane with thumbs down and the grading system was used in the version used in a previous study [25]. The ASES scores were also checked by the same physician and calculated as described in a previous study [26]. These outcome instruments were recorded before injection and at 3 weeks and 3 months after injection in the BMAC-PRP group, and before beginning exercise and at 3 weeks and 3 months after beginning exercise in the control group.

**Fig. 2:**
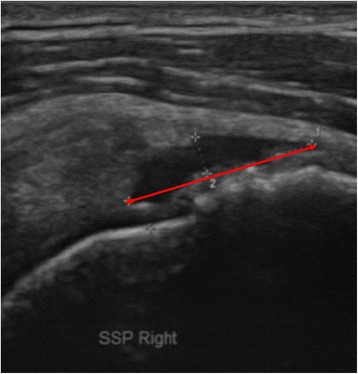
The sonographic image showed the greatest longitudinal tear along the long axis of the rotator cuff tendon and muscle in the modified Crass position. The tear size was measured by the greatest longitudinal tear length (red line) in several longitudinal sections of rotator cuff

Rotator cuff tear was evaluated with an ultrasound machine (Voluson^®^ E6, Siemens, Munich, Germany) equipped with a linear probe (Model No. 11L-D, 3.0-12.0MHz) at baseline and at 3 weeks and 3 months after the injection or exercise program. The ultrasound image of the rotator cuff tendon and muscle was selected that showed the greatest longitudinal tear size (mm) along the long axis of the rotator cuff tendon and muscle in the modified Crass position. The torn area was defined as hypoechogenic or anechogenic with irregular margins, and the tear size was measured by the greatest longitudinal tear length (mm) in several longitudinal sections of rotator cuff (Fig. 2). To prevent inter-observer variability [27], one experienced physiatrist who was blind to the group assignment performed all measurements of tear size.

Oral medication including non-steroidal anti-inflammatory drugs and pure pain-killers was allowed during the study period and change of the medication in frequency and dose was recorded before and 3 months after the procedure.

Mann-Whitney *U* tests or independent *t* tests were used to assess differences in change of VAS, ASES scores, MMT, and tear size between groups. Chi-square test was used to find the change of the medication between groups. SPSS 20.0 software (IBM Corp, Chicago, IL, USA) was used for the analysis and *P* values less than 0.05 were considered statistically significant.

**Fig. 3:**
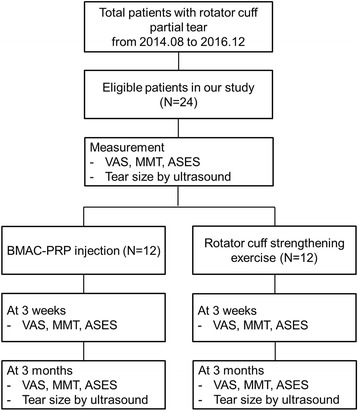
Flow diagram for non-randomized clinical trials was presented. Patients were assigned to the control group sequentially after recruitment was completed for the BMAC-PRP group

The protocol of this study was approved by the Institutional Review Board of Samsung Medical Center, and the patients were registered in the institutional board registry (registry number: 2014-07-173). Written informed consents were acquired from all patients. Flow diagram for non-randomized clinical trials was presented in Fig. 3.

## Results

Average age was 54.9 ± 7.6 years (5 men and 7 women) in the BMAC-PRP group and 59.6 ± 7.2 years (8 men and 4 women) in the control group, which was not significantly different (*P* = 0.414). Symptom duration was 7.3 ± 4.9 months in the BMAC-PRP group and 5.1 ± 4.4 months in the control group, which was not significantly different (*P* = 0.264). The initial VAS score did not differ significantly between the BMAC-PRP group (5.8 ± 1.9) and the control group (5.7 ± 1.6; *P* = 0.906). Manual muscle testing revealed grade 4 in four patients and grade 3 in three patients in the BMAC-PRP group, and grade 4 in one patient and grade 3 in two patients in the control group (*P* = 0.208). The initial ASES score was 39.4 in the BMAC-PRP group and 45.9 in the control group, which was not significantly different (*P* = 0.228). Tear size was 9.7 ± 3.4 mm in the BMAC-PRP group and 8.9 ± 5.0 mm in the control group (*P* = 0.178).

There was no patient who was lost to follow-up in both groups. All the patients in the control group performed the rotator cuff exercise daily without omission. Three patients in the control group felt pain during the strengthening exercise, so they increased the dose of the non-steroidal anti-inflammatory drug. Two patients in the BMAC-PRP group felt pain immediately after the injection, but the pain was subsided several hours after taking the non-steroidal anti-inflammatory drug.

The VAS scores in the BMAC-PRP group changed to 2.3 ± 0.8 at 3 weeks and 1.9 ± 0.7 at 3 months, while those in the control group changed to 3.6 ± 2.3 at 3 weeks and 3.7 ± 1.8 at 3 months. The change in the VAS score was significantly different between groups at 3 months (*P* = 0.039) but not at 3 weeks (*P* = 0.147).

On the MMT, six patients in the BMAC-PRP group and three patients in the control group were grade 4 at 3 weeks and at 3 months. All other patients had normal MMT (grade 5). The distribution of MMT grades did not differ significantly between groups (*P* = 0.206 at 3 weeks and *P* = 0.206 at 3 months).

The ASES score changed from 39.4 ± 13.0 to 54.5 ± 11.5 at 3 weeks and 74.1 ± 8.5 at 3 months in the BMAC-PRP group, and changed from 45.9 ± 12.4 to 56.3 ± 12.3 at 3 weeks and 62.2 ± 12.2 at 3 months in the control group. The change in the ASES score differed significantly between groups at 3 months (*P* = 0.011) but not at 3 weeks (*P* = 0.712).

In the BMAC-PRP group, six patients decreased their medication in frequency or dose, five patients did not change, and one patient increased their medication at 3 months. In the control group, two patients decreased their medication, seven patients did not change their medication, and three patients increased their medication at 3 months, which was not significantly different from the BMAC-PRP group (χ^2^ = 3.333, *P* = 0.189).

**Fig. 4:**
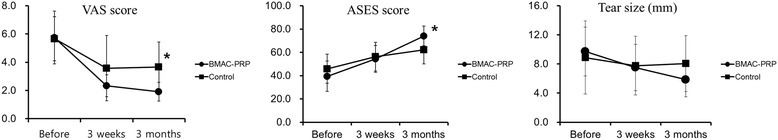
Changes in the VAS, ASES, and tear size between BMAC-PRP and control groups are expressed as mean ± one standard deviation. The changes in the VAS and ASES scores were significantly different between groups at 3 months but not at 3 weeks. BMAC-PRP decreased tear size, but this decrement did not show significant difference compared to the control group

The change in the tear size did not differ significantly between groups at 3 weeks or at 3 months (*P* = 0.235 and *P* = 0.312, respectively). All of the changes in the VAS, ASES, and tear size between groups are summarized in Fig. 4.

There were no side effects during bone marrow aspiration or injection of BMAC-PRP, and no complications in the follow-up period.

## Discussion

Our study showed that BMAC-PRP injection was associated with improved VAS and ASES scores at 3 months after injection as compared to the control group, while the change in the tear size and MMT did not differ between groups. There were no side effects or complications of BMAC-PRP injection.

We could not design this study as a double-blind study because bone marrow aspiration is an invasive procedure, and we could not discard BMAC after extracting it from the patients without use only for the purpose of blinding in the control group because of ethical issues. We recruited the BMAC-PRP group and control group sequentially instead of randomization because of the cost and safety issues. Bone marrow aspiration procedure is expensive and invasive, so we took up an insurance policy during the recruitment period of the BMAC-PRP group. This non-randomized design might affect our results, but we mitigated possible biases caused by non-randomized design by recruiting patients sequentially with the same indication for treatment and restriction as indicated by Cox et al. [28].

The VAS and ASES scores did not differ significantly between the BMAC-PRP and control groups at 3 weeks, but they were significantly different at 3 months. These results are consistent with the findings of another study, which found that subacromial autologous PRP injection in rotator cuff tear significantly improves pain and ASES scores at 12 weeks, but not at 6 weeks [29]. This 12-week effect was also found in a study by Scarpone that demonstrated that PRP led to improvement of pain and function 12 weeks after injection but not at 8 weeks after injection [30]. The exact reason for the effects of PRP on pain and function at 12 weeks is unknown. However, another study found that lameness of the tendon in tendinopathy started to decrease 8 weeks after PRP injection and higher performance occurred at 24 weeks, so at least 8 weeks may be necessary to show functional improvements with PRP [31].

We believe that one of the mechanisms for this pain reduction is downregulation of inflammatory mediators by PRP [32, 33] and BMAC [34]. We do not know whether PRP or BMAC has a greater impact on pain reduction; both may have played a role in reducing pain in our study. We did not assign the PRP group or BMAC group only to find the single effect of PRP or BMAC. Because the bone marrow aspiration procedure was an invasive procedure, we focused on the best way to acquire the maximal effect of possible therapeutic agents rather than to find a single effect of BMAC or PRP. Synergistic effects of the BMAC-PRP complex have been explored in previous studies [22, 35], which could be extrapolated to partial tear of the rotator cuff tendon in our thought. After confirming the therapeutic effect of BMAC-PRP complex on rotator cuff tears, we will have a plan to compare the effect of BMAC-PRP with that of BMAC or PRP.

We also found that shoulder function improved in the BMAC-PRP group at 3 months by increasing ASES scores. Several studies have reported that PRP or BMAC enhanced function of repaired rotator cuff tendons [36–39], which support our results. Although we did not investigate the histology of tendon tissues, this improvement in tendon function might be explained by the previous study which demonstrated the enhancement of tendon-bone junction healing by PRP [40].

In our study, BMAC-PRP did not decrease tear size. Decrease of tendon tear size is related to the proliferation of tenocytes and tendon stem cells and synthesis of collagen type III by tenocytes which happened during 6 weeks after the injury [41]. We could not investigate the collagen expression after BMAC-PRP injection by the tendon biopsy because of ethical issues, but BMAC-PRP might not enhance the synthesis of collagens to affect the tear size during 3 months. This was consistent with the previous study which demonstrated that BMAC-PRP did not show any significant difference in the expression of types I and III collagen synthesis [14]. The other explanation is small sample size to identify significant differences in tear size between groups. Establishing the effects of BMAC-PRP on tear size will require further studies with larger sample sizes.

The follow-up periods in our study were 3 weeks and 3 months, which was relatively short period considering that other surgical treatments for rotator cuff tear used 12 to 24 months follow-up period [38, 42]. However, Boorman demonstrated that outcome of non-operative treatment in chronic rotator cuff tear at 3 months could predict the success at 2 years after the initiation of treatment [43]. Therefore, we thought improvement of shoulder function at 3 months could suggest the success at 2 years after the injection, which might have an important meaning in the success of non-operative treatment in rotator cuff tears.

There were no side effects or complications during or after the injection. Pain evoked from the bone marrow aspiration procedure was not great enough to stop this study, and all patients in the BMAC-PRP group felt satisfied with the BMAC-PRP injection.

In this study, we could not design this study as a randomized controlled double-blind study because bone marrow aspiration is an invasive procedure. However, to confirm our results, a randomized controlled double-blind study will be necessary. The sample size in our study was 12 in each group, which was relatively small for a comparative study although sample size was calculated based on the statistical method. Further studies with a larger sample size will be necessary to clarify the therapeutic effects of BMAC-PRP on rotator cuff tears.

## Conclusions

This study found that BMAC-PRP improved pain and shoulder function in patients with partial tear of the rotator cuff tendon. Tear size decreased after BMAC-PRP injection although this decrement did not show any significant difference compared to the control group. A double-blind, randomized controlled study with a larger sample size will be necessary to clarify the therapeutic effects of BMAC-PRP on rotator cuff tear.

## Additional files

Additional file 1: Rotator cuff exercise comprised of stretching, scapular stabilization exercise, and strengthening exercise. (PDF 1536 kb)

## Abbreviations

ASES: American Shoulder and Elbow Surgeons
BMAC: Bone marrow aspirate concentration
BMMSCs: Bone marrow mesenchymal stem cells
MMT: Manual muscle test
PRP: Platelet-rich plasma
VAS: Visual analog scale
