# Supplementary material for: Effects of bone marrow aspirate concentrate and platelet-rich plasma on patients with partial tear of the rotator cuff tendon
Source: J Orthop Surg Res. 2018 Jan 3;13:1. doi: 10.1186/s13018-017-0693-x (PMC5753487; doi:10.1186/s13018-017-0693-x)
Supplement: Supplementary file 1 — Rotator cuff exercise comprised of stretching, scapular stabilization exercise, and strengthening exercise. (PDF 1536 kb) [file 13018_2017_693_MOESM1_ESM.pdf]

환자와 보호자를 위한 안내서

회전근개 증후군은 어떤 질환인가요?

happinnovation 20*20

SAMSUNG 삼성서울병원

목차

01 회전근개 증후군은 어떠한 질환인가요? 3

1) 어깨 관절과 회전근개란? 3

2) 회전근개 증후군이란 무엇을 말하나요? 4

3) 회전근개 증후군이 발생하고 악화되는 원인은 4

무엇인가요? 7

4) 회전근개 증후군의 증상은 무엇인가요? 6

5) 무엇을 해야 하나요? 6

6) 치료는 어떻게 해야 하나요? 7

01 회전근개 증후군은 어떠한 질환인가요?

1) 어깨 관절과 회전근개란?

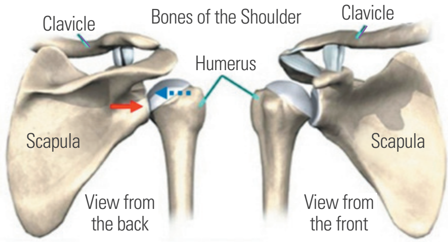

어깨 관절 (견관절) 은 우리 몸의 관절 중에 가장 큰 운동 범위를 갖고 있으며, 좌측 그림과 같이 견갑골의 관절 오목 부위에 상완골두 부위가 서로 마주 닿아 어깨 관절을 이룹니다. 관절 오목 부위의 면적이 작고, 상완골두 부위의 면적이 상대적으로 크기 때문에, 마치 조그마한 소켓에 큰 공이 붙어 있는 형태로 불안정한 구조물을 이루게 됩니다.

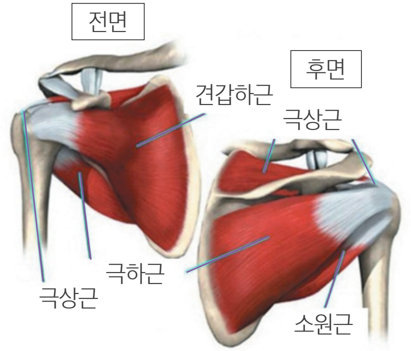

회전근개는 (rotator cuff) 이렇듯 불안정한 어깨 관절에 안정성을 제공할 뿐 아니라, 상완이 외전, 외회전, 내회전 할 수 있도록 움직여 주는 역할도 합니다.
회전근개는 극상근, 극하근, 견갑하근, 소원근의 네 가지 근육의 힘줄로 이루어지며, 견갑골에서 기시하여 상완골두에 부착합니다.
어깨 관절에서는 높은 기능적 운동이 이루어지므로 물리적 스트레스를 받기 쉽고, 많은 부하에 노출되기 때문에, 회전근개는 손상의 위험성이 높습니다. 즉, 반복적으로 미세손상을 입게 될 경우 회전근개 증후군으로 발전할 가능성이 있는 것입니다.

2) 회전근개 증후군이란 무엇을 말하나요?

회전근개 증후군은 하나의 질병으로 국한되는 개념이 아니며, 회전근개 퇴행 및 손상의 모든 구간을 포함하는 증후군입니다. 견봉하 충돌증후군 으로부터 회전근개 부분 혹은 전층 파열에 이르기까지 회전근개에 대한 일련의 손상 과정을 뜻합니다.

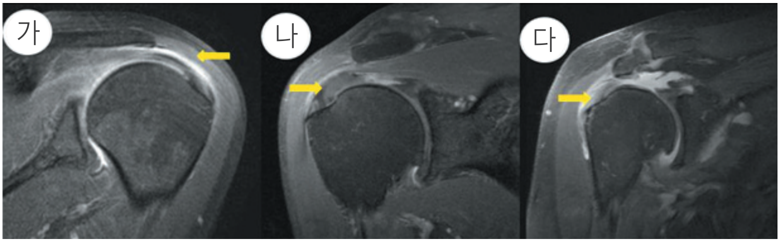

그림은 회전근개 증후군의 범주에 따른 어깨 자기공명영상 소견입니다.

- ‘가’ 는 견봉하 점액낭의 염증을 시사하는 소견이며, 회전근개 증후군의 비교적 초기단계부터 많이 관찰됩니다.
- ‘나’ 는 극상근 힘줄에 부종과 종창이 있는 극상근 건증 소견이며 악화될 경우 부분 혹은 전층 건 파열 단계로 진행할 수 있습니다.
- ‘다’ 는 극상근 힘줄의 전층 파열이 발생한 경우로 극상근이 관절와 부위로 후퇴되어 있으며, 근위축이 동반되는 회전근개 증후군의 종창역이라 할 수 있습니다.

3) 회전근개 증후군이 발생하고 악화되는 원인은 무엇인가요?

회전근개 증후군의 원인에 대해서는 수 많은 요인들이 거론되고 있지만, 아직 명확한 기전은 밝혀지지 않고 있습니다. 현재는 여러 외인성, 내인성 기전들이 복합되어 발생하는 것으로 추측하고 있습니다.

(1) 외인성 기전 (extrinsic mechanism)

외부의 압력과 전단력이 반복적으로 회전근개에 작용하여 힘줄에 손상을 준다는 개념입니다.

예) 외인성 충돌증후군

회전근개를 구성하는 근육 중 하나인 극상근은 견봉하 공간 (★) 및 오구견봉궁 (★) 하방으로 주행하여 상완골에 부착하게 되는데, 바로 이 주행 공간이 좁아져서 극상근 힘줄에 지속적인 압력과 손상을 주게 되며, 이를 일컬어 외인성 충돌증후군이라 부릅니다.

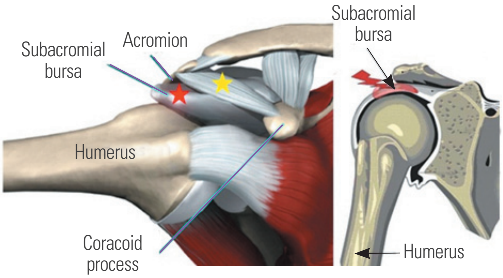

견봉하 공간을 좁게 만드는 원인은 첫째로 해부학적 요인이 있으며, 견봉하방과 견봉쇄골관절에 웃자란 뼈돌기가 존재할 경우, 견봉 모양 자체가 하방으로 돌출된 형태일 경우 등이 제기되고 있습니다. 둘째로 생역학적 요인이 있으며, 상완골의 골두가 주위 근육들의 부조화로 인하여 상방으로 이동하여 견봉하 공간이 좁아지게 되는 경우, 그리고 견갑골의 움직임에 이상이 생겨 견갑골의 후방 기울기와 상방 회전이 감소되는 경우가 거론되고 있습니다.

(2) 내인성 기전 (intrinsic mechanism)

외부적인 압력이나 전단력에 의해서가 아니라 회전근개 자체에 이상이 발생한다는 것으로, 가장 중요한 원인으로 '노화에 따른 힘줄의 퇴행성 변화' 가 있습니다. 회전근개 힘줄에 퇴행성 변화가 생길 경우 반복적인 미세손상에 취약하게 되어, 회전근개 증후군을 유발시킨다는 주장입니다. 이는 연령이 증가할수록 회전근개 증후군이 많이 발생하게 되는 근거가 됩니다. 퇴행성 변화 이외에 혈류 공급 이상이나 힘줄의 기질 성분 변화 등이 제기되고 있습니다.

4) 회전근개 증후군의 증상은 무엇인가요?

어깨 통증은 보통 서서히 발생하며, 윗팔 부위까지 확대되는 경우가 많습니다. 팔을 머리 위로 올리는 운동이나 자세를 취한 뒤에 통증이 악화되며, 보통 밤에 통증이 악화되어 자다가 깨기도 합니다. 회전근개 증후군이 진행되어 전층 파열이 있는 경우에는 팔을 옆으로 벌리거나 올릴 때 근력이 약화되어 있을 수 있는데, 머리를 빗을 때나 머리 드라이기를 사용할 때 어려움을 느끼게 됩니다. 또한 어깨 주위 근육이 위축되어 눈으로 보기에도 움푹 꺼진 양상이 관찰되기도 합니다.

5) 무엇을 해야 하나요?

회전근개 증후군의 증상이 있거나 점차 발생하고 있다면 전문의의 진찰을 받도록 합니다. 진찰시 먼저 어깨의 다양한 면의 관절운동범위를 측정하는데, 팔을 옆으로 벌리며 올릴 때 특정 각도에서 통증이 유발되는 지 여부를 확인할 것이며, 관절운동범위의 제한여부도 확인을 할 것입니다. 그리고 통증을 유발시키는 검사와 회전근개의 근력 검사를 시행하여 회전근개 증후군 여부를 확인하게 됩니다. 다음으로 어깨의 여러 관절에 퇴행성 관절염이나 석회 침착

등의 기저질환 여부를 확인하기 위해 방사선 촬영을 하게 되며, 회전근개의 손상 여부와 정도를 확인하기 위해 자기공명영상을 (MRI) 시행해야 하는 경우도 있습니다.

6) 치료는 어떻게 해야 하나요?

치료에서 가장 우선시 되어야 할 것은 통증을 유발시키거나 악화시키는 자세나 운동을 회피하여, 증상의 악화를 막는 것이 중요합니다. 팔을 머리 위로 올리는 자세는 피하는 것이 좋습니다. 수영, 배드민턴, 공 던지기와 같이 팔을 어깨위로 올리고 반복적으로 움직이는 운동은 좋지 않습니다. 또한 무거운 물건을 들 때에는 팔꿈치를 몸에 붙인 상태로 유지하는 것이 좋습니다.
통증으로 인하여 일상생활에 제약이 발생할 경우 약물치료를 받을 수 있습니다. 그리고 비정상적인 상완골과 견갑골의 움직임을 회복시키고, 약화된 회전근개를 강화시키기 위하여 운동치료가 병행되어야 합니다.
그럼에도 불구하고 통증이 지속될 경우 주사치료를 시행할 수 있습니다.
퇴행성 변화가 발생한 회전근개를 정상으로 되돌리기에는 힘들기 때문에, 치료 목표는 일상생활을 하는데 제약이 없을 정도로 통증을 경감시키고 악화되지 않도록 하는 데에 있습니다.

(1) 약물치료

보통 비스테로이드성 항염증제가 처방되는데, 회전근개의 자극과 염증을 줄여 통증을 경감시키는 역할을 합니다.

(2) 주사치료

초음파로 견봉하 점액낭의 위치를 확인한 후 스테로이드 제재와 국소 마취제를 혼합하여 주입합니다. 주사치료는 목적이 통증을 경감시키는데 있으며, 근본적인 치료제는 아니기 때문에, 주사 후 통증이 경감되어도 과도한 운동이나 통증을 유발시키는 자세는 피하여야 합니다.

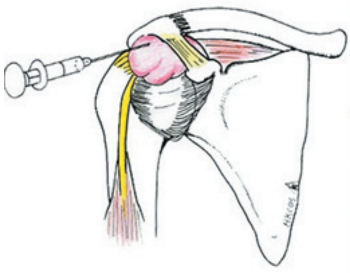

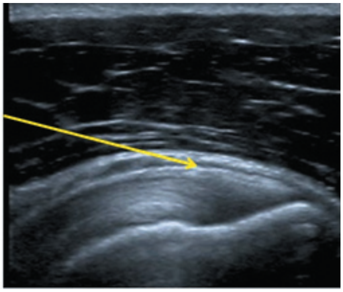

(3) 운동치료

운동치료 순서는 먼저 어깨 관절범위 운동과 스트레칭 운동으로 어깨 관절 가동 력을 정상화한 후 견관절 안정화 및 회전근개의 근력 강화 운동을 시행해야 합니다. 어깨 관절범위운동과 스트레칭 운동은 매일 시행되어야 하며, 근력강화 운동은 이틀에 한번씩 하는 것이 추천됩니다.
운동 치료는 병원 등 의료 기관에서 의료인에 의하여 시행되기도 하며, 의사 및 물리치료사를 통해 적절한 운동방법을 배운 뒤 가정에서 대부분의 운동을 스스로 시행할 수도 있습니다.
운동을 하기 전에 샤워를 하거나, 따뜻한 수건, 찜질팩을 이용하여 관절 주변을 따뜻하게 데워주면 연조직 및 주변 근육이 이완되어 운동을 하는데 도움을 줍니다. 아래의 운동을 통증이 느껴지지 않는 정도로 시행해야 합니다. 지속적으로 운동을 하여 어느 정도 익숙해지면, 근력 강화 운동을 강도와 저항력을 높혀서 시행하면 됩니다.

어깨 관절범위 운동 및 스트레칭 운동

흔들이 운동

어깨의 힘을 뺀 후 한쪽 손에 아령(또는 생수병)을 듭니다. 반대측의 손은 의자나 탁자에 붙히고, 상체를 지탱하면서 합니다.
전후, 좌우로 흔들고 원을 그리며 돌립니다.

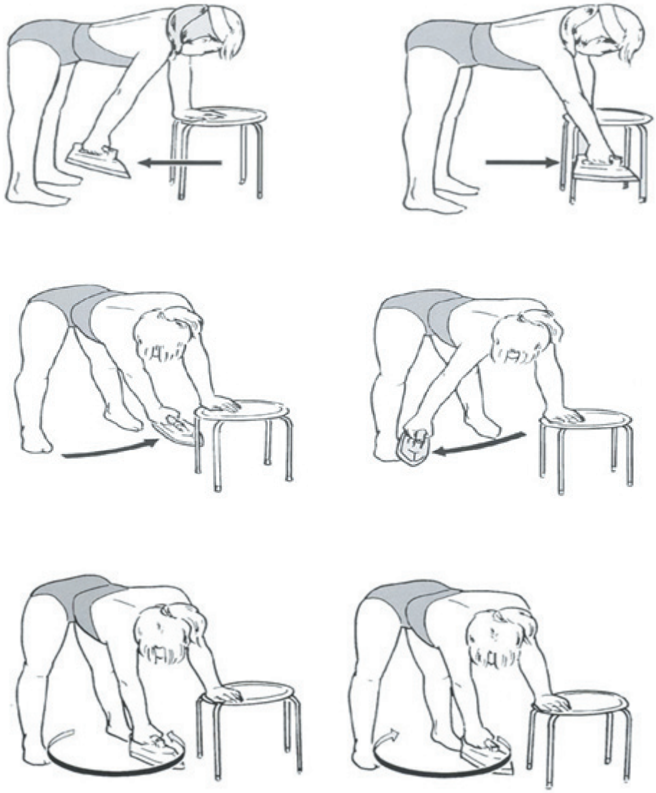

벽을 이용한 스트레칭운동

팔꿈치를 구부리고, 양손의 폭은 어깨 넓이 정도로 벌립니다. 손을 붙이고 상체를 앞으로 당깁니다. 벽을 미는 듯한 자세를 10-15초 간 유지합니다.

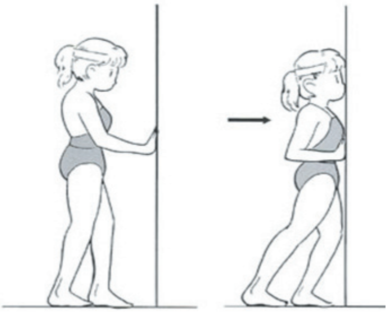

손을 조금 더 높은 위치에 놓을 수 도 있고, 문틈이나 벽의 모서리를 이용하여 같은 방법으로 스트레칭 운동을 해도 좋습니다.

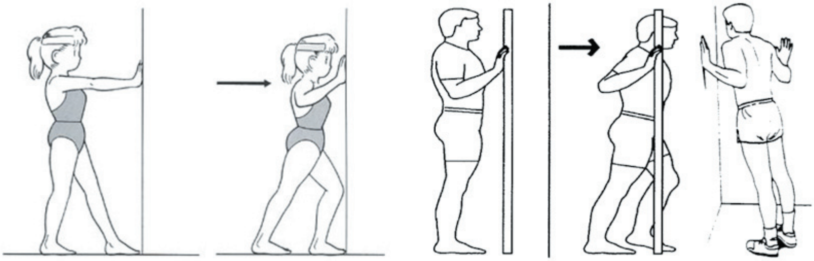

수건이나 봉을 이용한 관절범위운동

팔꿈치를 펴고 배쪽에서 가능한 한 위로(머리쪽으로) 수건을 들어올립니다. 처음으로 돌아가서 반복하십시오.

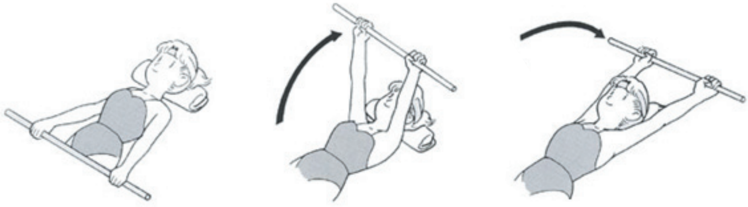

팔을 직각으로 올린 다음 좌측으로 움직입니다. 다시 처음으로 돌아가서 우측으로 움직입니다.

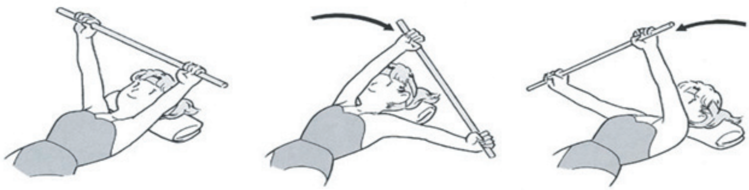

팔을 직각으로 올리고 좌우로 교차해서 비틀어 줍니다.

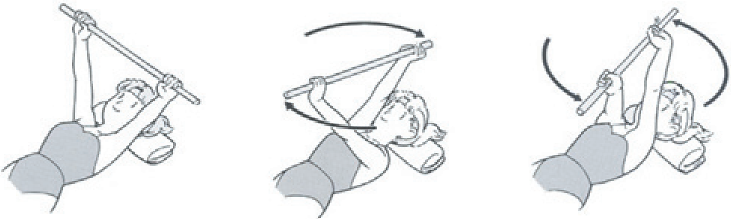

■ 견갑골 안정화 및 회전근개 근력 강화 운동

10회씩 반복하며, 운동 시 통증이 발생 또는 증가하면 운동 횟수를 줄여서 시행합니다. 또한 통증이 없는 정도의 운동 강도로 시행하여야 합니다.

견갑골 뒤로 기울이기 운동

어깨를 으쓱하며 견갑골의 윗부분을 뒤로 기울이는 운동을 합니다.
주먹을 쥐고 팔꿈치를 몸에 붙인 다음 견갑골의 윗부분을 뒤로 기울이며, 견갑골의 안쪽 면이 서로 마주 닿는 기분으로 운동을 합니다.
엉덩이에 양손바닥을 놓고 하셔도 됩니다.

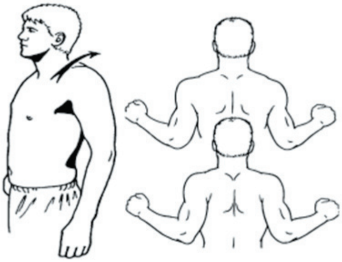

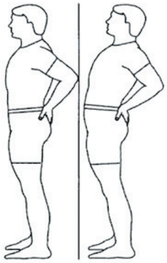

줄이나 탄력밴드를 이용한 하부 승모근 강화 운동

허리를 펴고 등이 의자에 닿지 않게 앉은 다음 양손에 줄이나 탄력밴드를 이용하여 잡아당기는 운동을 합니다.
10번 반복한 다음 1분 휴식하기를 3차례 시행합니다.

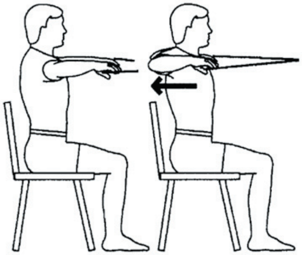

아래 그림과 같이 일어서서 해도 무방하며, 비슷한 원리로 아령을 사용하는 것도 가능합니다.

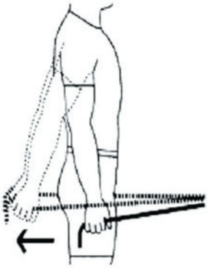

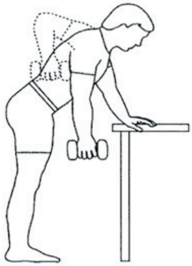

변형 팔굽혀 펴기

1번 그림처럼 팔굽혀 펴기 준비자세를 취합니다. 이후 2번 그림처럼 등을 새우등처럼 세우고 10초간 버티는 동작을 취합니다. 이런 동작을 반복하여 운동합니다.

1

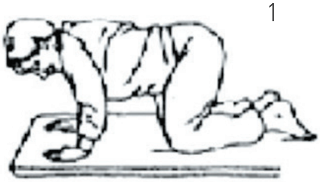

2

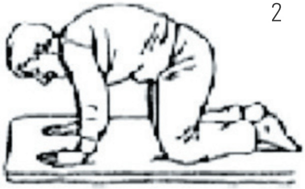

바깥방향 돌리기(외회전) 및 안쪽방향 돌리기(내회전)

A

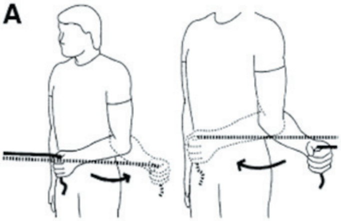

그림처럼 팔꿈치를 허리에 붙인 다음 고정하고, 탄력밴드를 이용하여 손을 바깥방향 또는 안쪽방향으로 돌리고 10초간 버티는 운동을 합니다. 10회 반복 후 1분 휴식하기를 총 3차례 시행합니다.

B

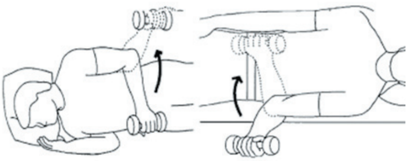

탄력밴드를 이용한 운동을 통하여 어느 정도 근력 강화가 되었다면, 아령을 이용한 근력강화 운동을 할 수 있습니다. 그림처럼 옆으로 누운 자세에서 팔꿈치를 붙인 다음 아령을 상방 및 하방으로 이동하기를 반복합니다. 10회 반복후 1분 휴식하기를 총 3차례 시행합니다.

“Copyright, http://www.eorthopod.com/ All rights reserved”
“Copyright, Kuhn JE. Exercise in the treatment of rotator cuff impingement. J Shoulder Elbow Surg. 2009 Jan-Feb;18(1):138-60. All rights reserved”
본문의 그림 및 일부 내용은 상기 문헌에서 인용하였으며, 이에 대한 모든 권리는 각각 상기 문헌과 저자에 있음을 밝혀 둡니다.

“Copyright, Department of Physical and Rehabilitation Medicine, Samsung Medical Center. All rights reserved”
상기 언급한 내용 이외의 본 책자의 모든 내용에 대한 권리는 Department of Physical and Rehabilitation Medicine, Samsung Medical Center 에 있으며 기타 명시되지 않은 모든 권리는 유보되어 있습니다.

REH-37-1503-2

환자와 보호자를 위한 안내서

회전근개 증후군은 어떤 질환인가요?

삼성서울병원
SAMSUNG MEDICAL CENTER

서울시 강남구 일원로 81 (우편번호 135-710)

대표번호 | 1599-3114 (예약, 안내)

홈페이지 | www.samsunghospital.com

※ 사전 허가 없이 본 책자 내용을 전재, 인용 및 각종 서비스 등에 사용하는 것을 금지합니다.
